# Maternal Syphilis in Mississippi, 2013 to 2023

**DOI:** 10.1001/jamanetworkopen.2025.46787

**Published:** 2025-12-30

**Authors:** Manuela Staneva, Victoria Gholar, Thomas Dobbs

**Affiliations:** 1 University of Mississippi Medical Center, John D. Bower School of Population Health, Jackson

## Abstract

This cross-sectional study evaluates patient characteristics and trend changes in Mississippi’s maternal syphilis rate from 2013 to 2023.

## Introduction

Maternal syphilis poses a substantial risk for catastrophic birth outcomes, such as preterm birth and neonatal death.^1^ Yet despite its severe consequences and preventable nature, maternal syphilis has reemerged in the US. Between 2016 and 2022, the maternal syphilis rate in the US increased by 222%, an uptrend that varied by state.^2^ Mississippi, for instance, exhibited the second highest rate of increase. To understand this unfolding public health crisis better, we described patient characteristics and evaluated trend changes in Mississippi’s maternal syphilis rate.

## Methods

This was a retrospective cross-sectional study of Mississippi’s 2013 to 2023 birth certificate data. Since 2013, Mississippi has been collecting information on maternal syphilis within this dataset under the variable “infections present and/or treated during this pregnancy.” The unit of analysis was births, and the study population included all Mississippi resident births. Maternal syphilis rates were calculated as the number of live births to women with syphilis per 100 000 live births. Race is self-reported in these data, and we defined urban status according to the National Centers for Health Statistics urban-rural classification scheme.^3^ The research was approved by the University of Mississippi Medical Center’s institutional review board and was exempted from the need for informed consent because it used deidentified data. This study followed the STROBE guidelines.

We performed descriptive analyses to highlight key patient characteristics. To examine temporal changes in the maternal syphilis rate from 2013 through 2023, we employed the National Cancer Institute’s Joinpoint Regression Program (version 5.4.0.0). This regression starts with a model containing no joinpoints (a straight line) and iteratively tests whether adding 1 or more joinpoints significantly improves the fit of the model.^4^ Each joinpoint denotes a statistically significant trend change, dividing a time series into straight lines (trend-segments). The analysis generates 2 useful statistics: the annual percentage change (APC) for each trend-segment and the average APC (AAPC) for the entire study period. Implementing weighted bayesian selection criterion, we selected the most parsimonious model at 2-sided *P* < .05.

## Results

Between 2013 and 2023, there were 1421 pregnant women with syphilis in Mississippi: 1281 (90.1%) were unmarried, 1057 (74.4%) were Black, and 679 (47.8%) were aged 24 years or younger (Table). Of the 1368 women with known prenatal care, 466 (34.1%) had no first trimester prenatal care and 61 (4.5%) had no prenatal care at all. The number of pregnant women with syphilis increased by 960.6%, from 33 in 2013 to 350 in 2023 (Figure, panel A). Exhibiting a similarly sharp upturn, the maternal syphilis rate increased 1088.0%, from 85.5 cases per 100 000 births in 2013 to 1015.7 cases per 100 000 births in 2023. The AAPC increase for the study period was 27.5% (95% CI, 25.7% to 32.2%; *P* < .001) (Figure, panel B).

**Table.** Demographic Characteristics of the Study Population

Characteristics	Participants, No. (%) (N = 1421)	Maternal syphilis rate, No. of cases/100 000 live births
Race		
Black	1057 (74.4)	619.8
Hispanic	29 (2.0)	146.3
Other^a^	36 (2.5)	342.1
White	299 (21.1)	154.2
Age group, y		
15-24	679 (47.8)	438.6
≥25	742 (52.2)	296.4
Residence^b^		
Metropolitan	653 (45.9)	335.1
Nonmetropolitan	768 (54.1)	366.4
Marital status		
Married	140 (9.9)	75.7
Not married	1281 (90.1)	583.3

^a^ Other race includes American Indian or Alaska Native, Asian, and Native Hawaiian or Other Pacific Islander.

^b^ Metropolitan areas contain an urban core with a population of 50 000 or more. Nonmetropolitan includes micropolitan with a core urban area of population less than 50 000 but more than 10 000 and noncore rural counties that lack a core urban area.

**Figure.**
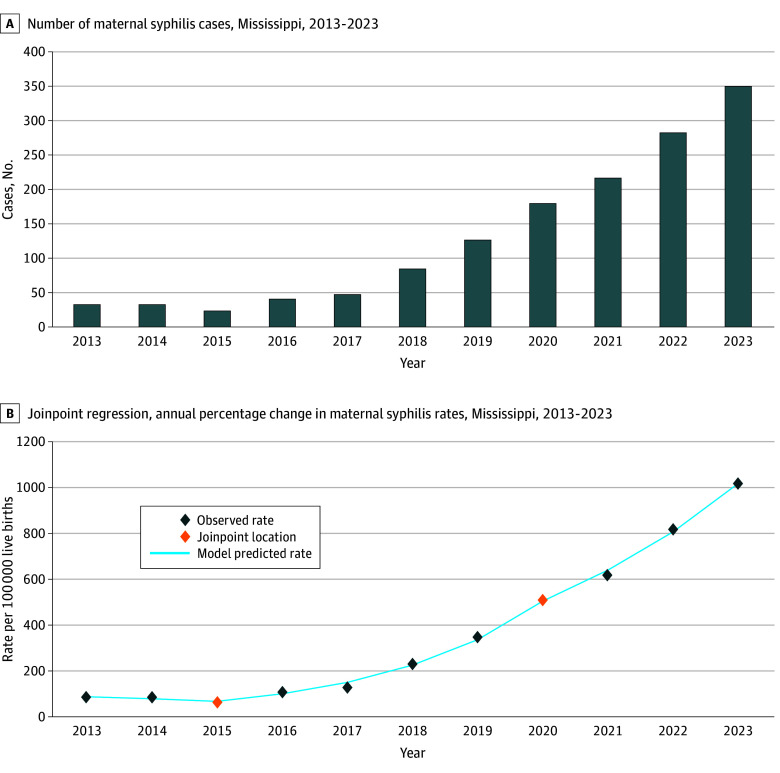
Maternal Syphilis in Mississippi, 2013 to 2023 A, Graph shows number of syphilis cases. B, Graph shows joinpoint regression annual percentage change (APC) in maternal syphilis rates. The orange diamonds indicate a joinpoint location. There were 3 trend segments: a nonsignificant decline in the trend from 2013 to 2015 (APC, −13.3%; 95% CI, −25.2% to 10.3%; *P* = .20) and significant trend increases during 2015 to 2020 (APC, 50.0%; 95% CI, 44.7% to 67.4%; *P* < .001) and 2020 to 2023 (APC, 26.3%; 95% CI, 20.1% to 32.4%; *P* < .001).

## Discussion

During 2013 to 2023, maternal syphilis was highly prevalent among young, single, and Black mothers in Mississippi with inadequate access to prenatal care. From a low plateau during the first 3 years of the period studied, maternal syphilis started to increase rapidly from 2016, a year marking the beginning of this maternal health crisis in Mississippi. This cross-sectional study expands on previous research and underscores the urgent need for improving access to prenatal care and providing adequate syphilis treatment to the most vulnerable populations in a state with limited resources, poor health care access, and deep socioeconomic inequities.^5^ Building a robust and sustained public health response to this epidemic has become, however, increasingly difficult because of chronic underfunding of syphilis prevention. Years of declining public health investments have left a dwindling core of public health nurses and disease intervention specialists, eroding the infrastructure that was previously in place to trace and treat syphilis.^6^ Although our research revealed the utility of birth certificate data for maternal syphilis surveillance, study limitations include a lack of information on maternal treatment and newborn syphilis status.

## Notes

Supplement. Data Sharing Statement
