# Supplementary material for: Maternal Syphilis in Mississippi, 2013 to 2023
Source: JAMA Netw Open. 2025 Dec 30;8(12):e2546787. doi: 10.1001/jamanetworkopen.2025.46787 (PMC12754680; doi:10.1001/jamanetworkopen.2025.46787)
Supplement: Supplement. — Data Sharing Statement [file jamanetwopen-e2546787-s001.pdf]

Data Sharing Statement

Staneva. Maternal Syphilis in Mississippi, 2013 to 2023. *JAMA Netw Open*. Published December 04, 2025. doi:10.1001/jamanetworkopen.2025.46787

Data
Data available: Yes
Data types: Data (not involving human participants)
How to access data: Manuela Staneva at manuela.staneva@umc.edu
When available: With publication

Supporting Documents
Document types: Statistical/analytic code
How to access documents: Manuela Staneva at manuela.staneva@umc.edu
When available: With publication

Additional Information
Who can access the data: All researchers interested in this study.
Types of analyses: Trend analyses
Mechanisms of data availability: Manuela Staneva at manuela.staneva@umc.edu
